# Identification of the Philadelphia-like subgroup in Turkish pediatric patients with acute lymphoblastic leukemia

**DOI:** 10.1007/s00277-026-06845-0

**Published:** 2026-02-25

**Authors:** Ecem Efendi Erdem, Gulsah Cecener, Ufuk Unal, Havva Tezcan Unlu, Melike Sezgin Evim, Birol Baytan, Unal Egeli, Yesim Oymak, Berrin Tunca, Adalet Meral Gunes

**Affiliations:** 1 Department of Medical Biology, Faculty of Medicine, Bursa Uludağ University, Bursa, Turkey; 2 Department of Paediatrics, Division of Paediatric Haematology and Oncology, Faculty of Medicine, Bursa Uludağ University, Bursa, Turkey; 3 Department of Pediatric Hematology and Oncology, Dr. Behçet Uz Pediatric Diseases and Surgery Training and Research Hospital, University of Health Sciences, İzmir, Turkey

**Keywords:** Philadelphia-like, Acute lymphoblastic leukemia, Pediatric, Gene expression profile, RT-qPCR

## Abstract

Ph-like ALL, a high-risk subgroup of B-cell ALL, is associated with a poor prognosis. The genetic diversity observed across different ethnicities underscores the importance of population-specific studies to gain a deeper understanding of its genetic drivers and clinical outcomes. This study aims to characterize the Ph-like ALL group in Turkish pediatric B-ALL patients and evaluate our custom-developed gene panel for subgroup identification. To identify the Ph-like subgroup, RNA was isolated from 35 bone marrow samples, and targeted mRNA expression analysis was performed by RT-qPCR using a custom-designed panel consisting of 96 genes. Additionally, the Archer FusionPlex ALL Panel (ArcherDX, Boulder, CO) was employed for further evaluation of patients demonstrating the highest similarity rates. This study employed a gene panel designed to differentiate Turkish Ph-like ALL patients within the Ph-negative group, identifying two Ph-like cases (6%). The panel demonstrated 96.9% sensitivity and 93.9% specificity in detecting Ph-like ALL, highlighting its effectiveness in differential diagnosis. In the Ph-like cases, alterations in *PAX5* (rs143723948, rs879020782) and *IKZF1* (rs6975767) were observed. Both cases shared a novel *EPOR* variant (rs1312770718), with no known clinical impact. Additionally, a novel variantin the *PTPN11* gene was interpreted as “Possibly Damaging”. This study introduces a gene profiling-based diagnostic approach for the Ph-like subgroup of pediatric B-ALL in Turkish patients. The integration of targeted tyrosine kinase inhibitors into treatment protocols, guided by the diagnostic algorithm, aims to improve prognosis and survival, advancing personalized management of Ph-like ALL.

## Introduction

Philadelphia-like acute lymphoblastic leukemia (Ph-like ALL) is a high-risk subtype of B-cell precursor ALL characterized by a transcriptional profile resembling *BCR-ABL1*–positive ALL (Ph-positive), despite lacking the classical *BCR-ABL1* fusion [1–4]. Large-scale genomic studies over the past decade have demonstrated that Ph-like ALL is driven by a heterogeneous spectrum of kinase-activating alterations converging on cytokine receptor and tyrosine kinase signaling pathways, thereby redefining its biological and clinical framework [5]. Seminal work identified recurrent, targetable lesions including ABL-class fusions, *JAK2* and *EPOR* rearrangements, and *CRLF2* overexpression, highlighting opportunities for precision therapy [6]. Studies in both pediatric and adult cohorts have demonstrated that detailed molecular profiling significantly improves diagnostic accuracy for this high-risk subtype [7–10].

Functional genomic investigations further revealed a broad distribution of clinically actionable kinase fusions across pediatric cohorts, strengthening the rationale for integrating targeted inhibitors (e.g., Imatinib, Dasatinib, Ponatinib, Ruxolitinib, Tofacitinib, Fedratinib) into therapeutic strategies [6, 9–13]. More recent studies from diverse geographic regions have demonstrated that the molecular landscape of Ph-like ALL exhibits population-specific variability, with distinct alteration patterns and adverse prognostic features, particularly in resource-limited settings [9, 10, 14]. These observations emphasize the necessity of population-adapted diagnostic strategies and cost-effective workflows, particularly in regions with limited access to advanced sequencing technologies [15, 16].

The identification of Ph-like ALL currently relies on a multimodal diagnostic approach that incorporates gene expression profiling, next-generation sequencing (NGS), fluorescence in situ hybridization (FISH), and fusion-directed RT-qPCR assays, enabling detection of transcriptional signatures and underlying kinase alterations [5–8, 11, 15]. Gene expression profiling remains the reference standard, while NGS and FISH complementarily detect structural variants and recurrent rearrangements, and targeted RT-qPCR provides a rapid and feasible screening tool for routine laboratories [5–8, 11, 15, 17].

Considering Turkey’s population genomic characteristics, their potential relevance to disease biology, and a relatively high prevalence of consanguineous marriages, investigating Ph-like ALL in Turkish patients is crucial for refining risk stratification and optimizing targeted therapeutic approaches. Considering known geographic and ethnic differences in the molecular landscape, characterizing Ph-like ALL in Turkish patients is essential for refined risk stratification, identification of actionable lesions, and optimized implementation of targeted therapies [18, 19]. This study addresses this gap by applying gene expression profiling and fusion-directed analyses in newly diagnosed pediatric B-ALL cases to establish a population-adapted diagnostic framework for Ph-like ALL.

## Materials and methods

### Patients characteristics

Between 2018 and 2020, 110 pediatric B-cell ALL cases were referred to our laboratory. According to the ethics-approved protocol, only treatment-naïve diagnostic bone marrow samples were eligible for gene expression profiling. A detailed review of clinical, immunophenotypic, and cytogenetic records revealed that 75 cases did not meet the inclusion criteria because the submitted samples were obtained at relapse, were not collected at initial diagnosis, or carried defining genetic abnormalities that place them outside the Ph-like ALL spectrum. These cases were therefore excluded. The final study cohort consisted of 35 cases, of which 2 were classified as Ph-positive and 33 as Ph-negative based on conventional molecular and cytogenetic analyses. The study was approved by the Medical Ethics Committee (2018-4/11) and adhered to the Helsinki Declaration.

### RNA isolation and RT-qPCR analysis

RNA isolation from bone marrow materials was performed using Trizol reagent (HibriGen, Turkey). All RNA samples were analyzed for quantity and quality using the DU-800 UV/Vis Spectrophotometer (Beckman Coulter, Brea, CA, USA), RNA purity verification was performed based on the 260/280 ratio, and an OD260/280 between 1.8 and 2.1 was considered indicative of RNA purity suitable for quantitative PCR analysis.

From the total RNA, RT-qPCR analysis was performed with the LightCycler^®^ EvoScript RNA Probes Master mix (Roche, Switzerland) protocol for gene expression testing. The prepared mixture was uploaded on a custom panel where the oligonucleotides of the genes were implanted. Expression differences at the gene level between the Ph-positive and Ph-negative patient groups of 96 different genes determined were evaluated. The custom panel, which was designed for the genes we identified to establish the differential diagnosis in the study, each consisting of 384 wells (RealTime ready Custom Panel 384 − 96/Roche, Switzerland), was arranged by the evaluation of 4 different patient samples. There are 93 genes and three housekeeping genes (*GAPDH*,* G6PD*,* ACTB*) for four different custom panels.

In accordance with MIQE guidelines, *GAPDH*,* ACTB*, and *G6PD* were selected as housekeeping genes. These reference genes have been widely validated as internal controls in international gene-expression classifiers for ALL and Ph-like ALL, including RT-qPCR–based diagnostic studies conducted in hematopoietic and leukemic tissues [12, 20, 21]. Given the potential variability of housekeeping gene expression in leukemic cells, a multi-gene normalization strategy was applied, whereby expression values were normalized to the geometric mean of *GAPDH*,* ACTB*, and *G6PD* to improve analytical stability and minimize sample-specific variation.

### Archer fusionplex ALL panels test

Targeted RNA sequencing for fusion detection was performed using the Archer^®^ FusionPlex^®^ ALL Panel (ArcherDX, Boulder, CO, USA) according to the manufacturer’s recommendations. Total RNA extracted from diagnostic bone marrow samples was quantified and assessed for integrity before library preparation. The assay is based on anchored multiplex PCR (AMP) technology, which enables unbiased detection of gene fusions without requiring prior knowledge of fusion partners.

Library preparation involved the following steps:

(i) cDNA synthesis from total RNA using reverse transcription;
(ii) end repair and adapter ligation with molecular barcodes that facilitate error correction and duplicate read removal;
(iii) two-step PCR enrichment, where gene-specific primers targeting exons frequently involved in ALL-associated rearrangements were used in combination with universal adapters to amplify both known and novel fusion events.

Prepared libraries were quantified, normalized, and sequenced on an Illumina platform using paired-end reads following standard Archer workflows [22].

Raw sequencing data were analyzed using the Archer Analysis software, which performs read alignment, molecular barcode–based error correction, deduplication, and fusion calling. Detected fusions were evaluated based on read depth, breakpoint confidence, and supporting unique start sites, and only high-confidence calls were included in the final interpretation.

### Statistical analysis

Gene expression data and clinical characteristics of patients were compared using the independent t-test and the chi-square test. According to the quantitative results obtained, SPSS 25.0 and GraphPad Prism 8.1 (USA) programs were used for all statistical analyses. Relative gene-expression levels were calculated using the 2^(-ΔΔCt) method. ΔCt values were obtained by subtracting the geometric mean Ct of the housekeeping genes (*GAPDH*,* ACTB*,* G6PD*) from the Ct of each target gene, and ΔΔCt values were calculated using the Ph-negative, non–Ph-like group as the reference. All analyses were performed at a 95% (95% CI) confidence interval, and *p* < 0.05 was considered statistically significant. Although no universally accepted cutoff exists, Ct values around 35 are generally considered to be at the threshold of detection in RT-qPCR analysis, indicating low target gene expression levels, and are often deemed borderline for reliable detection [12]. Consequently, Ct values exceeding 35 were excluded from the analysis, as they are associated with reduced sensitivity and potentially unreliable results. G*Power software v.3.1.9.7 was used to assess the power of the number of patients included in the analysis.

## Results

### Patients’ clinical profile

According to the study’s inclusion criteria, 35 bone marrow samples were collected diagnosed with precursor B-cell ALL (pediatric group under 18 years of age) who applied to the Department of Pediatric Hematology between 2018 and 2020 and had not received prior treatment. Gene expression findings of 33 Ph-negative cases were compared with 2 Ph-positive cases to identify the Ph-like group. Sample size calculations were performed using G-Power version 3.1.9.7, which determined a required sample size of 51 for both the Ph-negative and Ph-positive control groups, with a 95% confidence interval, a 5% margin of error, a large Cohen’s d effect size (0.5), and 80% power. In the study due to the focus on a pure group, the final sample consisted of 35 patients.

When the distribution of clinical findings in the patient profile is examined, the age range of pediatric cases was between 2 and 17, with a mean age of 7 years and eight months. Sixteen of the cases included in our study were female (51.6%), and 15 of them were male (48.3%). The cases were evaluated according to their clinical data based on the classification criteria in the ALL IC-BFM 2009 protocol, and 9.1% of the cases included in the study were classified as a standard-risk, 66.6% as an intermediate-risk, and 24.2% as a high-risk group (Table 1). The mean peripheral blood leukocyte (WBC) value was 10.7 K/µL, hemoglobin (HGB) levels were 11.1 g/dL, and platelet (PLT) levels were 113.3 K/µL at the time of diagnosis. The Minimal residual disease (MRD) analysis was carried out at the Institute of Human Genetics at Heidelberg University Hospital.

**Table 1:** Clinical characteristics of patients

Patients	Age of Diagnosis	WBC	HGB	PLT	MRD	Treatment Response	Relaps
**BUU-01**	15 years 7 months	6,08 K/µL	9,82 g/dL	43,4 K/µL	-	Ex	**✓**
**BUU-02**	2 years	8,53 K/µL	11,30 g/dL	55,2 K/µL	-	Ex	**✓**
**BUU-03**	5 years	3,43 K/µL	13,10 g/dL	41,7 K/µL	Good	Good	-
**BUU-04**	10 years	27,9 K/µL	7,56 g/dL	56,8 K/µL	Good	Good	-
**BUU-05**	9 years	27,1 K/µL	8,86 g/dL	62,8 K/µL	Unknown	Good	-
**BUU-06**	5 years	3,19 K/µL	9,38 g/dL	95,5 K/µL	Good	Good	-
**BUU-07**	9 years	2,44 K/µL	8,36 g/dL	73,2 K/µL	Good	Good	-
**BUU-08**	3 years	6,25 K/µL	11 g/dL	83,3 K/µL	Unknown	Good	-
**BUU-09**	12 years	8,88 K/µL	6,51 g/dL	72,6 K/µL	Poor***	Poor	-
**BUU-10**	4 years	2,99 K/µL	11,02 g/dL	95,63 K/µL	Unknown	Good	-
**BUU-11**	6,5 years	3,2 K/µL	9,1 g/dL	35,49 K/µL	Good	Good	-
**BUU-12**	4 years	4,35 K/µL	10,7 g/dL	27 K/µL	Unknown	Poor	-
**BUU-13**	3 years	4,06 K/µL	9,43 g/dL	21,41 K/µL	Good	Good	-
**BUU-14**	3 years 6 months	5,53 K/µL	7,61 g/dL	43,7 K/µL	Unknown	Good	-
**BUU-15**	3 years	21,5 K/µL	9,71 g/dL	60,4 K/µL	Good	Good	-
**BUU-16**	5 years 6 months	9,68 K/µL	9,7 g/dL	112,7 K/µL	Good	Good	-
**BUU-17**	5 years	5,82 K/µL	6,1 g/dL	43,4 K/µL	Unknown	Good	-
**BUU-18**	3 years	34,12 K/µL	7,5 g/dL	192,3 K/µL	Middle	Good	-
**BUU-19**	3 years	39,43 K/µL	9,8 g/dL	22,9 K/µL	Good	Poor	-
**BUU-20**	6 years	2,23 K/µL	10,1 g/dL	126,3 K/µL	Middle	Middle-Ex	**✓**
**BUU-21**	2 years	19,22 K/µL	12,1 g/dL	100,3 K/µL	Poor***	Ex	**✓**
**BUU-22**	17 years	2,18 K/µL	8,15 g/dL	31,23 K/µL	Unknown	Middle	-
**BUU-23**	12 years	3,58 K/µL	10,2 g/dL	69,1 K/µL	-	Good-Ex (Infection)	-
**BUU-24**	8 years	20,13 K/µL	12,3 g/dL	62 K/µL	Poor***	Poor	**✓**
**BUU-25**	5 years	**144,9 K/µL	4,1 g/dL	14,7 K/µL	Good	Middle	-
**BUU-26**	2 years	*56 K/µL	8 g/dL	55,8 K/µL	-	out of follow-up patient	-
**BUU-27**	3 years 10 months	29,60 K/µL	5,8 g/dL	30 K/µL	Good	Good	-
**BUU-28**	8 years	12,38 K/µL	12,08 g/dL	65,6 K/µL	Good	t(1;19) HR	-
**BUU-29**	2 years 3 months	*54,88 K/µL	7,7 g/dL	138 K/µL	Middle	Poor	-
**BUU-30**	2 years 9 months	5,07 K/µL	7,4 g/dL	77,4 K/µL	Good	Good	-
**BUU-31**	4 years 8 months	4,04 K/µL	7,6 g/dL	232,8 K/µL	Unknown	Good	-
**BUU-32**	17 years	**115 K/µL	5,22 g/dL	31,5 K/µL	Unknown	Good	-
**BUU-33**	16 years	10,7 K/µL	14,8 g/dL	107 K/µL	Unknown	Good	-

*WBC > 50 K/µL is the lower threshold usually used for clinically high risk in leukemia studies

**WBC > 100 K/µL is highly correlated with Ph-like ALL and is associated with poor prognosis

*** One of the cases with poor MRD response associated with poor prognosis and relapse risk was identified as Ph-like by our diagnostic algorithm

Bursa Uludağ University Health Application and Research Center Hospital Medical Biochemistry Laboratory Tests Minimum and Maximum Reference Values WBC: 4.5–11 K/µL, HGB:12.5–16.5 g/dL, PLT: 145–400 K/µL

MRD: MRD was assessed according to the ALL IC-BFM 2009 protocol. MRD <10⁻⁴ was defined as negative, and MRD ≥10⁻⁴ as positive. Treatment response was defined based on protocol-specific early response criteria, integrating MRD results and clinical response parameters

MRD was evaluated according to the ALL IC-BFM 2009 protocol at predefined treatment time points. MRD levels < 10⁻⁴ were defined as MRD-negative, while levels ≥ 10⁻⁴ were considered MRD-positive. Treatment response was classified based on early response criteria outlined in the protocol, incorporating MRD status and clinical parameters to stratify patients into response categories.

## Patients’ genomic landscape

The cases included in the study were classified into Ph-positive and Ph-negative subgroups based on cytogenetic analyses. Ph-positive cases were designated as the control group, and their gene expression profiles were compared with those of Ph-negative cases. Within the Ph-negative group, cases exhibiting expression profiles similar to the control group were defined as Ph-like ALL. In the cases identified as Ph-like ALL, other genetic alterations were assessed using Archer FusionPlex ALL Panel and MLPA analyses.

## Gene expression analysis

**Fig. 1:**
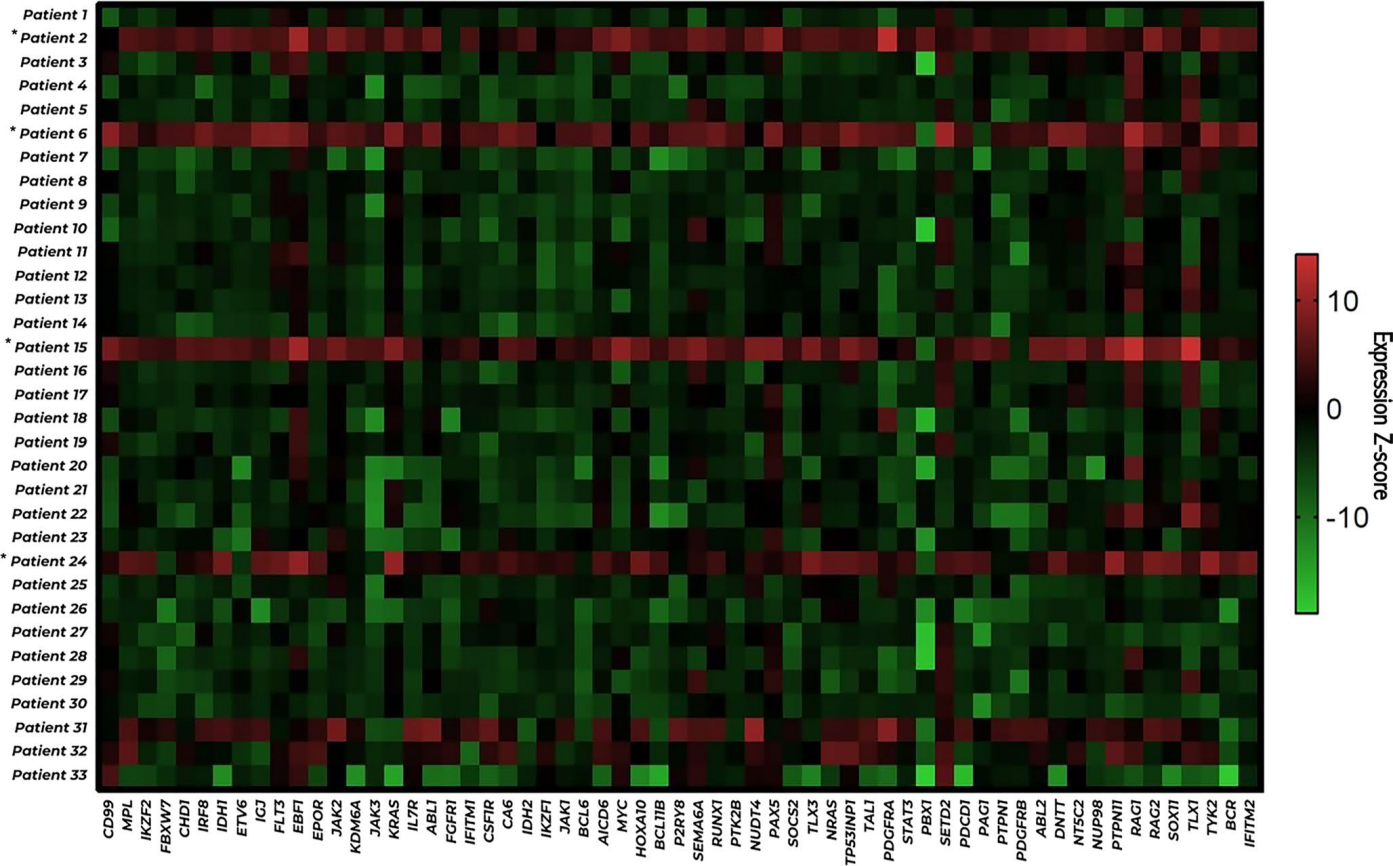
Heatmap visualization of normalized mRNA expression levels calculated using the ΔCt method for 61 genes with measurable expression derived from the initial 96-gene RT-qPCR panel in 33 clinically classified Ph-negative patients. Rows represent individual patients and columns represent genes. Color intensity reflects relative expression levels, with red indicating higher and green indicating lower expression. Patients 2, 6, 15, and 24 were identified as Ph-like ALL based on similarity algorithm–based analysis

The first analysis step in line with the study’s hypothesis was to determine the rate of similarity between the Ph-positive and Ph-negative groups. The gene expression analysis values obtained from the cases in two different groups were examined among the 96 genes analyzed by the RT-qPCR method, and the “Delta CT Method” was used to evaluate the similarity rates of 61 genes whose expression was determined (Fig. 1).

Statistical significance in our study confirms the “no difference” hypothesis. In the patients analyzed in our study, mRNA expression levels of 54 genes out of 61 genes were found to have expression levels similar to the Ph-positive group. The genes with the highest similarity frequency are respectively *P2RY8*,* NT5C2*,* SEMA6A*,* JAK2*,* RUNX1*,* FLT3*,* SETD2*,* TLX1*,* RAG1*,* RAG2*,* CD99*,* CA6*,* DNTT*,* TYK2*,* ABL2*,* ABL1*,* MYC*,* PAX5*,* PTPN11*,* PAG1*,* SOX11*,* AICDA*,* TP53INP1*,* KRAS*,* IRF8*,* IKZF1*,* STAT3*,* MPL*,* KDM6A*,* NUDT4*,* NRAS*,* TAL1*,* CHD1*,* IFITM2*,* PTPN1*,* ETV6*,* IDH2*,* EBF1*,* PBX1*,* IDH1*,* JAK1*,* HOXA10*,* FGCDR1*,* TLB3 BCR*,* IL7R*,* SOCS2*,* PDGFRA*,* IFITM1*,* EPOR*,* IGJ*,* NUP98*,* CSF1R*,* IKZF2*,* BCL6*,* BCL11B*,* PDGFRB*,* FBXW7*,* JAK3.* These genes are predicted to be potential biomarkers in identifying cases of Ph-like ALL in our study group.

**Fig. 2:**
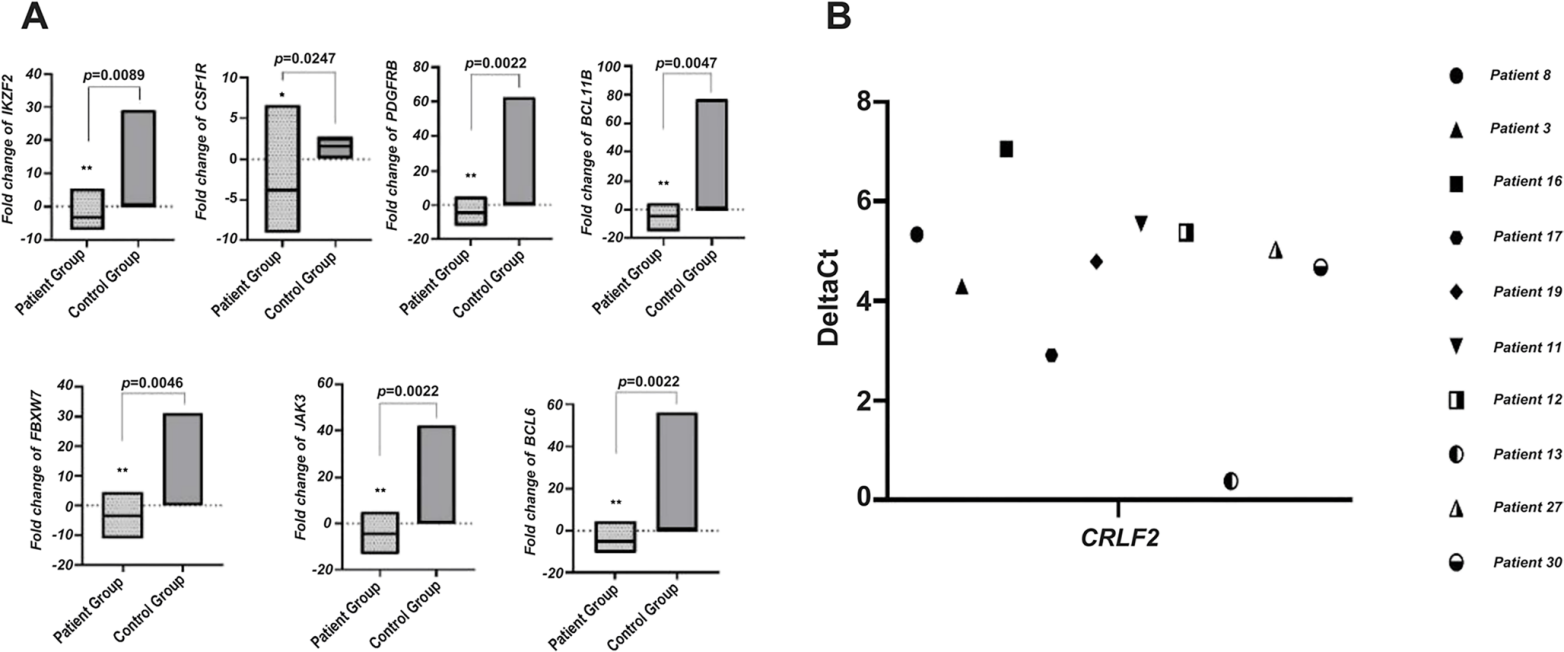
(**A**) Differential expression of selected genes showing statistically significant differences between the patient group (Ph-negative patients) and the control group (Ph-positive patients) (*; *p* < 0.05, **;*p* < 0.01) (**B**) *CRLF2* gene expression levels in individual patients are shown as ΔCt values, with each symbol representing a single patient, illustrating the inter-patient variability and highlighting cases with *CRLF2* overexpression relevant to the Ph-like ALL phenotype

A statistically significant difference was found in the mRNA expression levels of 7 genes with the data obtained from mRNA expression profiles in which Ph-positive and Ph-negative groups were compared (*p* < 0.05) (Fig. 2A). It is stated that approximately 50% of the Ph-like ALL patient group has a *CRLF2* rearrangement. Among the 33 patients included in our study, the *CRLF2* value of 23 patients could not be obtained. Therefore, *CRLF2* expression was evaluated in 10 patients, and all had elevated *CRLF2* expression (over expression) (Fig. 2B).

### Development of the Ph-like similarity algorithm

**Fig. 3:**
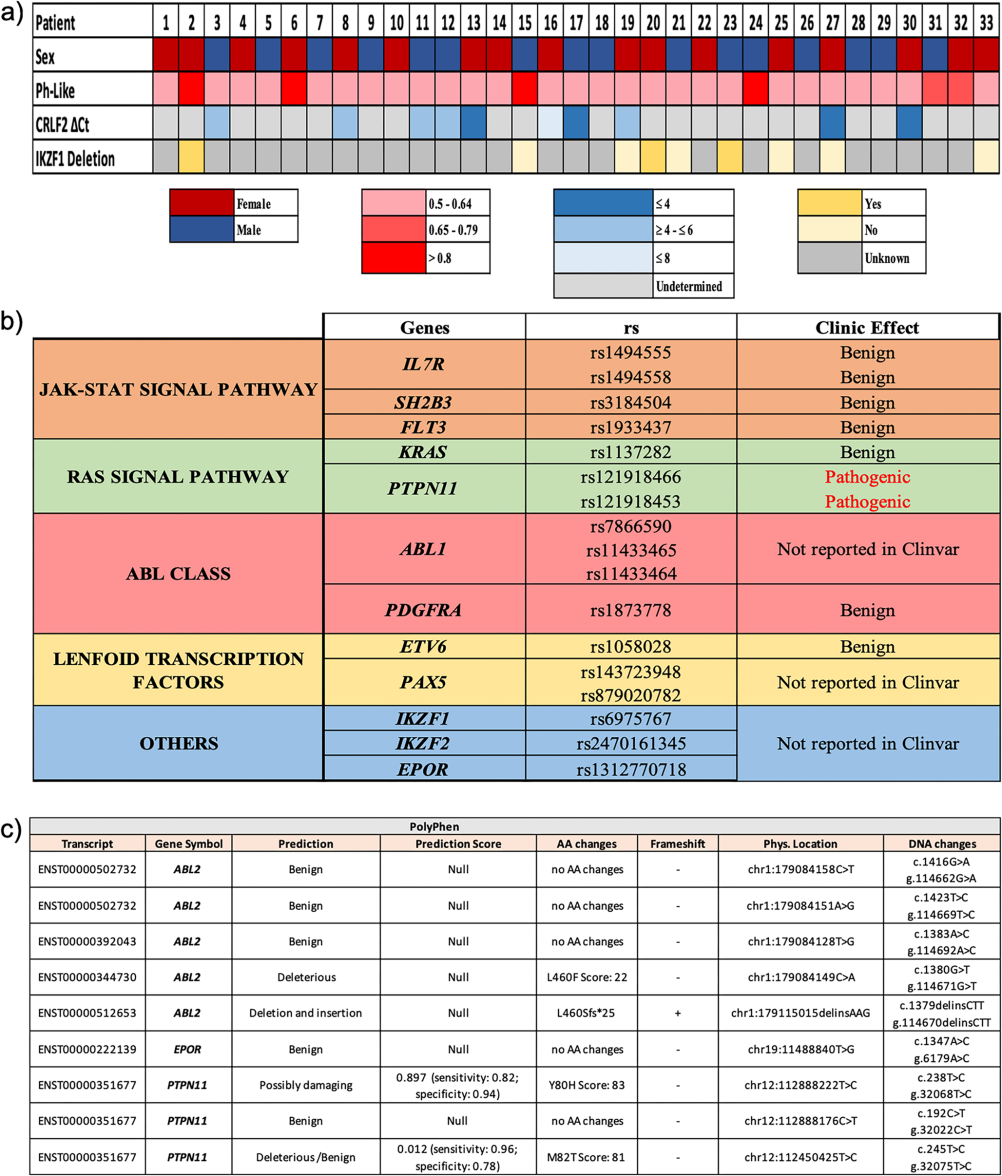
Molecular characterization of patients with the highest similarity **a**) Clinical and molecular features of the study cohort, including sex, Ph-like status, *CRLF2* expression levels, and *IKZF1* deletion status, are summarized **b**) Genetic alterations identified in the two patients (Patients 2 and 24) exhibiting the highest similarity based on gene expression profile analysis are shown. Following completion of the stepwise diagnostic algorithm, both cases were classified as Ph-like acute lymphoblastic leukemia (ALL). Genetic alterations commonly observed in Ph-like cases were categorized according to their associated signaling pathways and mechanisms of action, and their potential clinical implications were assessed **c**) Novel genetic alterations shared by the two Ph-like cases were further evaluated using the PolyPhen bioinformatics tool. One alteration was identified as novel and predicted to be potentially pathogenic

Expression data were analyzed with the delta CT model. The percentages of similarity between the patient groups were evaluated according to the fold regulation values obtained. First of all, expression findings of Ph-positive and Ph-negative groups were subjected to a normal distribution test. The cases were compared with the t-test to determine the difference between the Ph chromosome-positive cases. A statistically significant difference between the two groups was accepted as *p* < 0.05. The data providing the value of *p* > 0.05 were considered similar between the two groups. A degree of similarity was established according to the amount of greatness at *p* > 0.05. The results obtained determined that 4 (2nd, 6th, 15th, and 24th) patients had the most similar mRNA expression profile to the Ph-positive group (Fig. 3a).

### Genomic characterization and identification of Ph-like ALL patients

After the evaluation of gene expression profiles in Ph-like ALL, other specific genetic alterations are investigated. The custom gene panel demonstrated a sensitivity of 96,9% and a specificity of 93.9% in detecting Ph-like ALL, underscoring its effectiveness in the differential diagnosis of this subgroup. These specific alterations include ABL-class fusions, JAK-STAT signaling pathway alterations, other kinase and cytokine receptor pathway mutations. When the expression profiles of Ph-positive and Ph-negative cases were compared, four cases (12.1%) showed 85–95% similarity. However, only two of these cases carried Ph-like–associated genetic alterations confirmed by Archer FusionPlex ALL Panel analysis and MLPA, including kinase-activating lesions characteristic of the subgroup. The other two cases, despite showing partial transcriptomic resemblance, did not exhibit any Ph-like–defining structural variants or pathway-activating mutations, and therefore were not classified as Ph-like.

### Kinase alterations in Ph-like ALL (Fusion analysis)

The Archer FusionPlex ALL kit was used to effectively identify kinase fusions in all cases regardless of *CRLF2* expression. The rearrangements are seen in some of our cases; *KLF15-SOX11*, *ATP5L-KMT2A*, *LPP-BCL6*, *KMT2A-MLLT1*, *ETV6-RUNX1*. However, ABL class fusion changes were not observed in both cases defined as Ph-like.

### Sequence mutations and deletions in Ph-like ALL patients

Mutations activating transcription factors and signaling pathways in Ph-like ALL were identified by Archer FusionPlex analysis. In our two Ph-like patients, common mutations were detected in the *IL7R*,* SH2B3*,* FLT3*,* KRAS*,* PTPN11*,* ABL1*,* ABL2*,* PDGFRA*,* ETV6*,* PAX5*,* IKZF1*,* IKZF2* and *EPOR* genes (Fig. 3b). In addition to the *IKZF1* deletions and mutations identified in our Ph-like patients, alterations in other lymphoid transcription factors together suggest a high risk of relapse. Among the novel variants evaluated by PolyPhen analysis, the c.238T > C (p.Phe80Leu) change in *PTPN11* gene was interpreted as “Possibly Damaging” (Fig. 3c). Both pathogenic *PTPN11* variants identified in our cases demonstrated high-confidence sequencing metrics and in silico evidence of functional impact. The c.236 A > G (p.Gln79Arg) variant (rs121918466) was detected at an allele fraction of 0.0811, supported by a read depth of 185× and a high Phred quality score of 535.8; computational predictions indicated pathogenic potential, with a deleterious SIFT score (0) and a PolyPhen-2 score suggestive of a possibly damaging effect (0.844). Similarly, the c.214G > T (p.Ala72Ser) variant (rs121918453) was observed at an allele fraction of 0.0818 with 159× read depth and a Phred-scaled quality score of 452.6, accompanied by in silico predictions consistent with a deleterious (SIFT 0.02) and possibly damaging (PolyPhen-2 0.873) functional effect. For both alterations, COSMIC and FATHMM annotations currently classify their functional significance as unknown.

## Discussion

Ph-like ALL is recognized as a high-risk subtype with inferior treatment outcomes across pediatric and adult groups [19, 21]. Although gene expression–based classifiers have been widely investigated, their clinical utility remains limited, emphasizing the need for accessible and population-specific diagnostic tools [22–24]. In addition to gene expression profiling, recent international studies have introduced advanced classification methodologies-including targeted RNA-sequencing fusion assays (NARASIMHA), rapid molecular classifiers (PHi-RACE), surrogate-marker–based screening, and cost-effective diagnostic workflows such as the PACE approach-which collectively highlight ongoing efforts to refine differential diagnosis [25, 26].

**Fig. 4:**
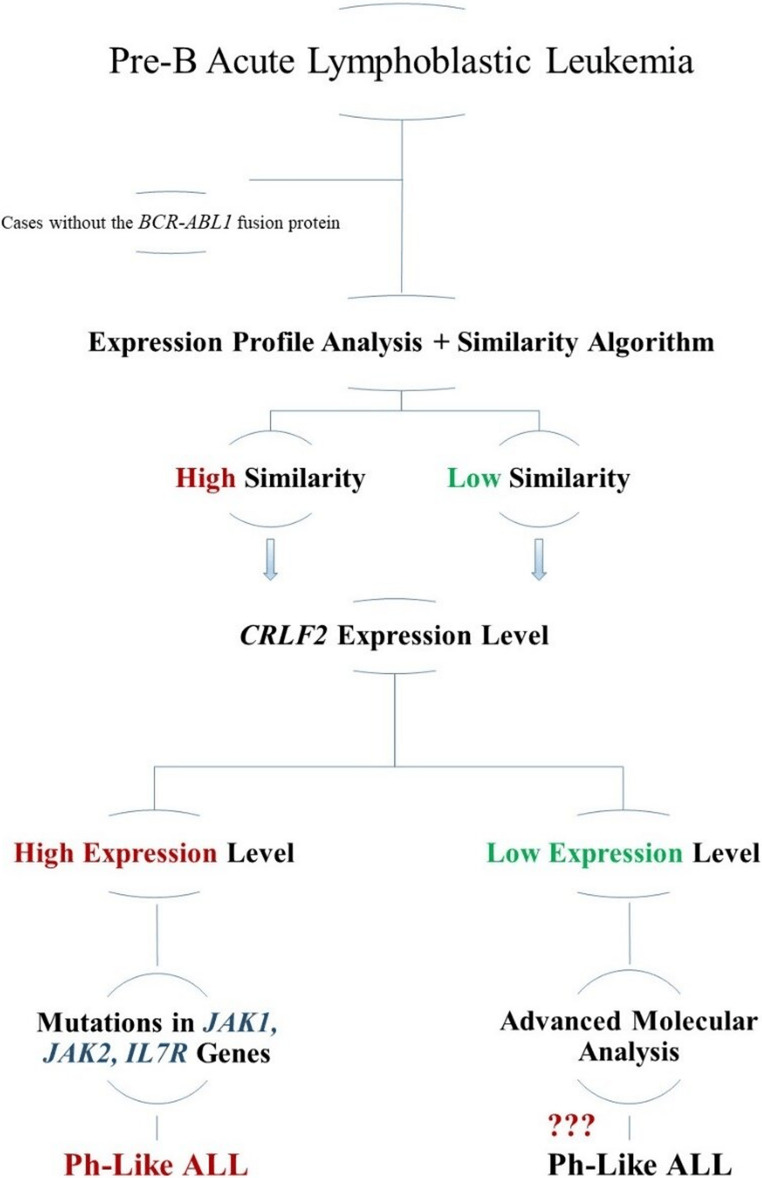
Progressive diagnostic algorithm for the identification of Ph-like acute lymphoblastic leukemia (ALL) Ph-like ALL cases, characterized by the absence of the *BCR–ABL1* fusion, require a multistep diagnostic approach due to their heterogeneous genetic background. In this stepwise algorithm, cases are initially stratified based on gene expression profile analysis combined with a similarity-based classification into high- and low-similarity groups. *CRLF2* expression levels are subsequently evaluated to further refine subgroup classification and guide the identification of underlying genetic alterations, including mutations in *JAK1*,* JAK2*, and *IL7R*. Cases lacking elevated *CRLF2* expression undergo additional molecular analyses to uncover alternative genetic drivers associated with the Ph-like ALL phenotype

This study aimed to develop a gene expression panel and diagnostic algorithm for identifying Turkish pediatric Ph-like ALL cases (Fig. 4), thereby providing insights into the epidemiological and molecular characteristics of Ph-like ALL within this population and evaluating a custom-designed gene expression panel specifically tailored to the distinctive genetic background of Turkish pediatric patients. A standardized diagnostic strategy for Ph-like ALL is still lacking, and classification often varies depending on the methodology used. The disease frequently involves kinase-activating alterations such as ABL-class fusions, *CRLF2* overexpression, JAK–STAT pathway mutations, and RAS/MAPK activation, the identification of which is essential for risk stratification and therapeutic planning. Ph-like ALL is characterized by targetable kinase-activating alterations, and selected subgroups—particularly those harboring ABL-class fusions—may benefit from TKI based therapy. The absence of uniform diagnostic criteria contributes to variation in prevalence and outcomes across studies, underscoring the importance of integrated molecular profiling [13, 17, 18].

Clinical presentation alone is often insufficient to classify Ph-like cases, as age at diagnosis and elevated WBC values may overlap with other ALL subtypes [21, 27, 28]. Genetic alterations, MRD response, and treatment outcomes represent more reliable diagnostic determinants [29]. In our study, WBC values and age criteria did not reach statistical significance, so genetic findings and MRD results were prioritized.

While previously published studies and commercial assays provide valuable gene sets for identifying Ph-like ALL, the marked genetic heterogeneity of this subgroup and the known variability of gene-expression signatures across different ethnic backgrounds indicate that GEP alone is insufficient for definitive classification [30]. In line with reports demonstrating population-related differences in GEP-based clustering, we evaluated these gene sets within the Turkish pediatric population and optimized a panel that showed the highest diagnostic relevance in our cohort.

Therefore, the panel is ‘specific’ not in the sense of ethnicity-driven unique variants, but in that it was empirically tested and refined in the Turkish population to ensure accurate pre-screening prior to targeted genomic analyses.

A major limitation of the present study is the relatively small number of cases included. Only 35 treatment-naïve diagnostic samples without alternative defining genetic lesions were available between 2018 and 2020. Larger, multicenter cohorts are essential to validate the performance of the proposed gene-expression panel and to more comprehensively characterize Ph-like ALL in the Turkish population. In this regard, efforts are currently underway to establish nationwide collaborations and to collect standardized diagnostic materials through pediatric leukemia associations across Türkiye.

According to research studies, it is stated that there are deletions in the genes that are effective in the B cell development stages of ALL cases that progress with a poor prognosis. When genes (*IKZF1*,* PAX5*,* E2A TCF3*,* EBF1*, and *VPREB1*) implicated in B cell development were examined for focal deletions, it was determined that one or more deletions occurred in 82% of the Ph-like cases.

The *IKZF1* gene, encoding the crucial transcription factor IKAROS, is deleted or mutated in 15% of pediatric B-ALL cases, 70% of Ph-positive cases, and 40% of Ph-like cases. These mutations are associated with treatment resistance [27]. *PAX5* mutations, present in 32% of Ph-like cases, contribute to both leukemia progression and drug resistance [31–34]. Alterations in *IKZF1* and *PAX5* are often observed together and are linked to poor prognosis and early relapse [27, 33]. In our Ph-like cases, mutations in *PAX5* (rs143723948, rs879020782) and *IKZF1* (rs6975767) were identified (Fig. 3b).

*CRLF2* rearrangements or point mutations are seen in about half of all cases of Ph-like ALL [2, 3, 35, 36]. *P2RY8-CRLF2* fusion is more common in standard-risk cases, and *IGH-CRLF2* fusion is more common in high-risk patients [37]. *CRLF2* expression was evaluated in Ph-negative patients included in our study, and *CRLF2* expression was found to be high in 30.3% of them (Overexpression ΔCt) (Fig. 2B). Expression values ​​of the *CRLF2* gene could not be obtained in other patients, including two cases defined as Ph-like ALL.

The second most common alteration in the Ph-like ALL subgroup is ABL class fusions (*ABL1*,* ABL2*,* CSF1R*,* PDGFRα*, and *PDGFRβ*) [27, 28]. ABL class fusions occur in 10% of Ph-like pediatric cases and 15% of adult cases. Research indicates that ABL class alterations are found in 45% of cases with low *CRLF2* expression, while they are present in only 5% of cases with high *CRLF2* expression [3, 27, 31, 32, 34].

In our study, although the expression values of the *ABL1* (*p* = 0.649), *ABL2* (*p* = 0.64), and *PDGFRα* (*p* = 0.078) genes were similar to the Ph-positive group, the *CSF1R* (*p* = 0.031) and *PDGFRβ* (*p* = 0.018) genes were statistically significant. Furthermore, the *CSF1R* gene was 3-fold down-expressed in the Ph-negative patient group, whereas the *PDGFRβ* gene was 4-fold down-expressed. Mutation analysis of ABL class genes in our Ph-like patients revealed common variants, which are shown in Fig. 3b. ABL class fusions were observed in 15.1% of our patients; however, kinase-activating rearrangements could not be detected. Additionally, in cases with *CRLF2* overexpression, ABL class alterations could not be assessed.

The interleukin 7 receptor (*IL7R*), encoded by the *IL7R* gene, plays a crucial role in lymphocyte development. Insertions or deletions in *IL7R* activate the JAK/STAT signaling pathway [34, 36]. In the study by Reshmi et al. [8], *IL7R* variants were identified in 173 of 284 Ph-like B-ALL cases. *SH2B3* variants, present in 91% of Ph-like ALL patients, were also observed in our cases. Two benign *IL7R* variants (rs1494558, rs1494555) and one benign *SH2B3* variant (rs3184504) were identified in common among two of our Ph-like cases (Fig. 3b).

The *EPOR* gene encodes the erythropoietin receptor, part of the cytokine receptor family. Erythropoietin binding to the *EPOR* stimulates *JAK2* tyrosine kinase, activating intracellular signaling pathways (RAS/MAPK, PI3K, and STAT) [3, 37]. *EPOR* is overexpressed in approximately 1% of Ph-like ALL cases. In our cohort, *EPOR* expression levels were similar to the Ph-positive group (*p* = 0.063). A common variant, rs1312770718, was identified in the two Ph-like cases, with no clinical significance reported (Fig. 3b).

*FLT3*-activating variants are present in 3.9% of Ph-like ALL cases [38]. A common benign variant (rs1933437) in the *FLT3* gene was identified in our two Ph-like cases (Fig. 3b).

Alterations in the RAS pathways are less frequent in Ph-like ALL, but these alterations are more frequently associated with other factors such as JAK-STAT pathways and *CRLF2*. RAS pathway mutations, including those in *KRAS*, *NRAS*, and *PTPN11*, may be critical for understanding the behavior of certain disease subgroups. The *KRAS* (rs1137282) and *PTPN11* variants common to our two Ph-like cases are shown in Fig. 3b. Among the five *PTPN11* variants identified, two were pathogenic (rs121918466, rs121918453), while the clinical impact of the remaining three variants is unknown.

Somatic mutations in the *IKZF2* and *ETV6* genes are frequent in B-ALL patients [39]. In the two Ph-like cases, *IKZF2* and *ETV6* variants were identified (Fig. 3b). The clinical significance of the *IKZF2* variant (rs2470161345) remains unknown, while the *ETV6* variant (rs1058028) is benign. Alterations in *IKZF2* are associated with low hypodiploidy B-ALL [36], but hypodiploidy was not observed in our study.

Novel variants detected in the studied genes were analyzed using PolyPhen. The c.238T > C (p.Phe80Leu) variant in *PTPN11*, identified in two Ph-like patients, was classified as “Possibly Damaging” with a score of 0.897, indicating that this change likely impairs protein function. No information regarding this variant was found in the ClinVar and gnomAD databases. This variant, located in the N-SH2 domain of *PTPN11*, causes a codon change from Phenylalanine to Leucine, potentially altering SHP2 protein conformation and increasing phosphatase activity. Gain-of-function variants in *PTPN11* are linked to hematologic malignancies, including juvenile myelomonocytic leukemia and some acute lymphoblastic leukemia cases. Based on these findings, the c.238T > C (p.Phe80Leu) variant is considered “novel” and “potentially pathogenic”. Further studies in larger patient cohorts are needed to clarify its association with the disease and integrate this variant into the literature (Fig. 3c).

According to clinical classification, 27.2% of the cohort met high-risk criteria, 9% were categorized as intermediate-risk, and 63.6% as standard-risk. Relapse occurred in 15.1% of patients, with an associated mortality rate of 80% among relapsed cases. Genetic assessment identified two patients with Ph-like ALL, both of whom were clinically assigned to the standard or intermediate-risk groups. A 5-year longitudinal follow-up is planned to evaluate event-free survival and associated genomic outcomes.

Ph-like profiles, determined by gene expression, vary across ethnicities. Our findings support the need for a community-specific diagnostic panel, with ongoing studies aimed at improving the reliability and specificity of our diagnostic algorithm. Novel alterations identified by the Archer FusionPlex ALL kit in our cohort offer promising insights.

In developing our diagnostic approach, canonical fusions (*BCR-ABL1*, *ETV6-RUNX1*, *TCF3-PBX1*, *KMT2A-MLL*) were excluded, and this study represents the first analysis of 96 genes in Turkish pediatric ALL aimed at delineating the Ph-like subgroup. The resulting 54-gene panel and bioinformatic algorithm may contribute to future modification of treatment protocols by integrating targeted agents such as tyrosine kinase inhibitors (imatinib, dasatinib, ruxolitinib), histone deacetylase inhibitors (chidamide), and bispecific antibodies (blinatumomab) with the goal of improving survival outcomes [40].

Ph-like ALL is defined by kinase pathway–activating alterations, rendering it amenable to TKI therapy, though clinical benefit varies by mutation type, co-occurring lesions, and patient age. The COG AALL1131 trial reported superior event-free survival with TKI therapy-particularly dasatinib-whereas *CRLF2/JAK2* cases derived no benefit [17, 19, 41]. Similarly, the St. Jude Total Therapy 17 study showed improved disease-free survival in ABL-class fusion cases treated with imatinib or dasatinib, and MD Anderson reported ponatinib efficacy in TKI-resistant *ABL1* T315I-mutated disease [42]. TKIs improve MRD negativity, reduce relapse risk, and provide better survival compared to chemotherapy. JAK inhibitors, such as Ruxolitinib, should be prioritized for *JAK2/CRLF2* alterations [43].

Ph-like ALL represents only 10–15% of B-ALL even in large multicenter cohorts, and the limited number of cases in our patient group likely reflects this low prevalence. Consequently, the molecular findings reported here should be considered preliminary, providing the first foundational data for the Turkish population.

In light of these findings, this study highlights the utility of a targeted gene expression panel for identifying Ph-like ALL in Turkish pediatric patients and supports the integration of multimodal molecular diagnostics to improve risk stratification. The identification of novel and potentially pathogenic variants in *PTPN11* and other signaling pathways provides avenues for future research and may ultimately inform precision-guided treatment approaches in this population.

## Conclusion

Preliminary observations from this cohort suggest that Turkish pediatric patients may exhibit population-specific genetic features that could influence mutation frequency and treatment response. To validate these findings, future clinical trials and multi-institutional studies will be essential for refining patient selection, evaluating the utility of TKI-based therapeutic approaches, and advancing standardized precision-oncology guidelines.

## Data Availability

Data cannot be shared openly, to protect the privacy of study participants as agreed with them in the informed consent but can be made available by email to anyone wishing to write to us and keep this confidential.
